# Bronchonodal fistula: An uncommon complication of tuberculosis

**DOI:** 10.1590/0037-8682-0127-2025

**Published:** 2026-06-15

**Authors:** Edson Marchiori, Bruno Hochhegger, Alexandre Dias Mançano, Gláucia Zanetti

**Affiliations:** 1 Universidade Federal do Rio de Janeiro, Departamento de Radiologia, Rio de Janeiro, RJ, Brasil.; 2 University of Florida, Department of Radiology, Gainesville, FL, USA.

**FIGURE 1:**
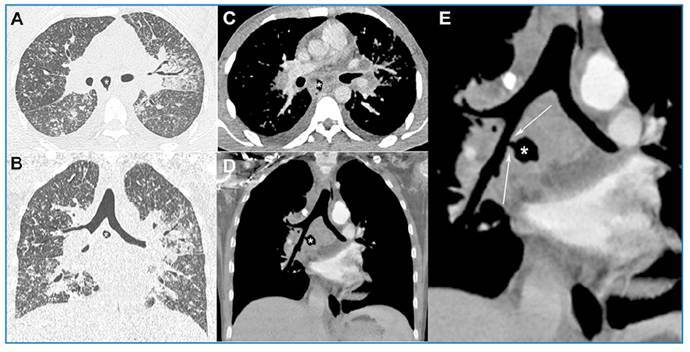
Axial **(A)** and coronal **(B)** chest computed tomography (CT) scans with pulmonary windows show micronodular infiltrate, paracardiac consolidations, and an air cavity of subcarinal localization (asterisk). Axial **(C)** and coronal **(D)** CT scans with mediastinal windows and detail **(E)** show enlarged subcarinal lymphadenopathy with necrosis and the fistulous trajectory of the air cavity (necrotic lymph node), draining to the bronchus intermedius (arrows in **E**).

A 25-year-old male presented with a five-month history of dyspnea, fever, productive cough, and weight loss. Physical examination and laboratory test results were unremarkable. Chest radiography revealed non-homogeneous consolidations and micronodular infiltration. Chest computed tomography (CT) showed micronodular infiltrate, paracardiac consolidations, and a subcarinal air cavity (necrotic lymph node), with a fistulous trajectory draining to the bronchus intermedius (Figure 1). Bronchoscopy revealed a focal mucosal defect covered with necrotic tissue in the bronchus intermedius, corresponding to the location of the bronchial fistula on CT. There was caseous material draining into the bronchus. A sputum Xpert study and culture were positive for *Mycobacterium tuberculosi*s. A final diagnosis of tuberculosis was established. The patient was placed on a six-month antituberculosis treatment regimen, and clinical improvement and marked radiological regression were noted at the last follow-up evaluation.

Lymph-node tuberculosis is the most common form of extrapulmonary tuberculosis. In the thorax, tuberculous mediastinal lymph nodes may enlarge, partially or fully obstructing the airway. They can also erode adjacent structures such as the heart, aorta, pleural space, and esophagus, forming fistulas and causing fatal consequences. Airway erosion may cause intrabronchial spread, leading to distal airspace nodules or consolidation^1^ ^-^ ^4^. Bronchoscopy is essential to confirm the diagnosis, although some fistulas are not readily visible because their tracts are covered by exudate or necrotic tissue^3^. Thus, CT is an important tool to confirm the fistulous trajectory, airway compromise, and parenchymal complications.
